# Diffuse hydralazine-associated vasculitis with pathergy: An overlapping picture of Sweet syndrome and bullous vasculitis

**DOI:** 10.1016/j.jdcr.2023.07.026

**Published:** 2023-08-01

**Authors:** Sara Banbury, Corinne Rauck, Eric E. Morgan, Maria F. Arisi, Juliana Berk-Krauss, Timothy Buckey, Olajumoke Fadugba, Misha Rosenbach

**Affiliations:** a Perelman School of Medicine at the University of Pennsylvania, Philadelphia, Pennsylvania; b Department of Dermatology at University of Pennsylvania, Philadelphia, Pennsylvania; c Department of Pathology & Laboratory Medicine, Hospital of the University of Pennsylvania, Philadelphia, Pennsylvania; d Division of Pulmonary, Allergy and Critical Care, Perelman School of Medicine, University of Pennsylvania, Philadelphia, Pennsylvania

**Keywords:** ANCA-positive vasculitis, hydralazine-associated vasculitis, hydralazine vasculitis, hereditary alpha-tryptasemia

## Introduction

Hydralazine is an antihypertensive associated with drug-induced systemic lupus erythematous (SLE). Hydralazine-associated vasculitis has limited reports in the literature.^1^ These reports include an association with characteristic cutaneous findings of acral hemorrhagic pseudoembolic vesiculopustules with necrosis and mucosal involvement.^2^ Here, we present a case of hydralazine-associated bullous vasculitis with overlapping findings of Sweet syndrome.

## Case report

**Fig 1:**
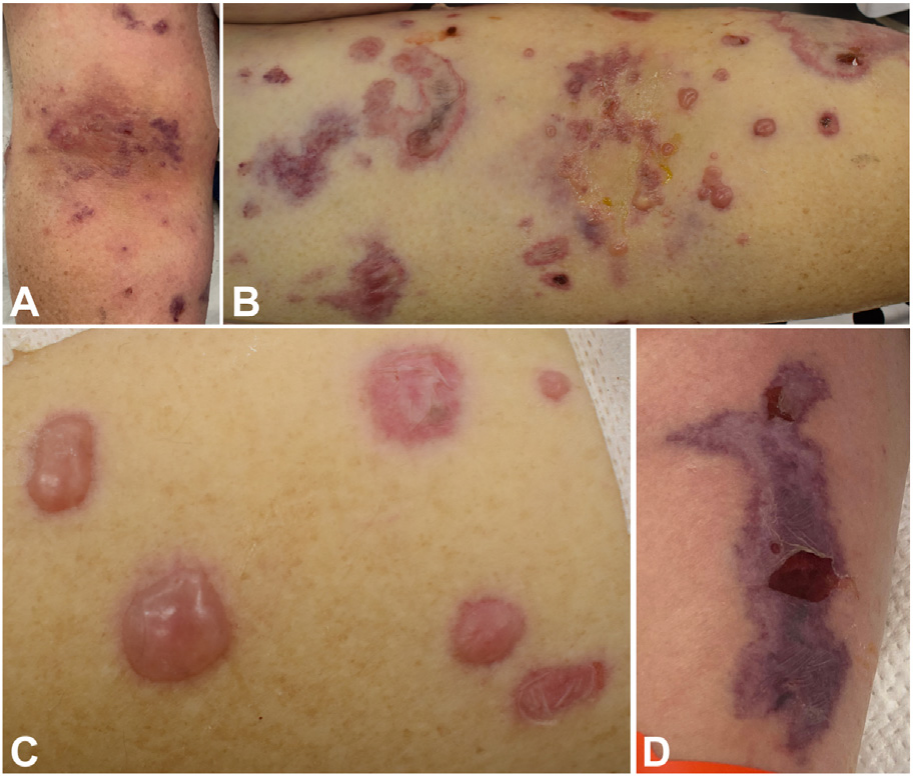
Dermatologic findings of (**A**) early right antecubital fossa, (**B**) late right antecubital fossa, (**C**) left forearm, and (**D**) right forearm.

**Fig 2:**
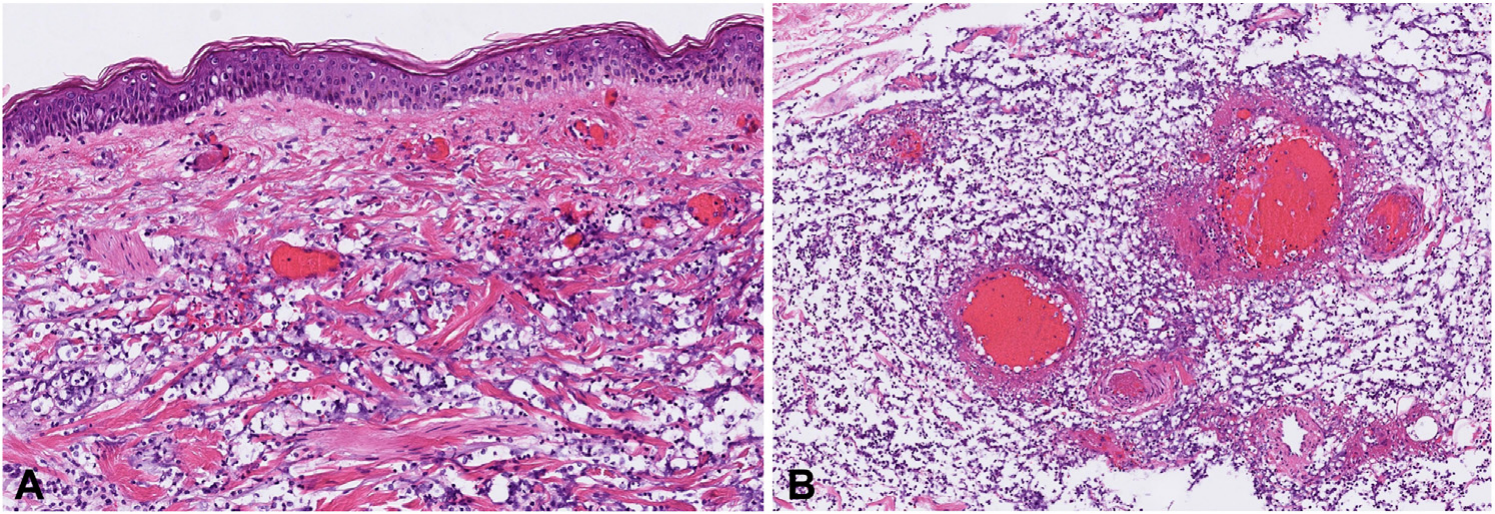
**A,** Postmortem skin specimens demonstrate a brisk dermal infiltrate composed of “Cryptococcoid” mononuclear cells with perinuclear clearing; (**B**) Subcutaneous medium-vessel vasculitis with prominent mononuclear infiltrate. (**A** and **B,** Hematoxylin-eosin stain; original magnifications: **A,** 200×; **B,** 400×.)

A white woman in her 60s with a medical history notable for hypertension, long COVID-19, and hereditary α-tryptasemia (HaT) initially presented with a subarachnoid hemorrhage secondary to hypertensive urgency. Her medications while hospitalized included clonidine, famotidine, fexofenadine, loratadine, metoprolol, and montelukast. Her home hydralazine, which she had been on for many years, had been stopped 2 weeks before presentation, although she received 2 doses while admitted. While hospitalized, she developed clustered hemorrhagic vesicles and tense bullae, including in areas of trauma (eg, extremities at phlebotomy and arterial line sites, lip under endotracheal tube), consistent with pathergy (Fig 1). Her rash morphology also included purpuric patches and plaques on her extremities. Dermatology was consulted, and a punch biopsy from a purpuric lesion demonstrated dermal thrombosis with features of acute tissue injury and interstitial neutrophilic inflammation (Fig 2). PAS and Gram stains were negative. Tissue culture for bacteria, atypical mycobacteria, and fungus did not show evidence of infection. Varicella-zoster virus and herpes simplex virus PCR swabs from the base of intact vesicles were negative. The lesions did not progress over the next 48 hours. She did have a white blood cell count elevated to 12.1 THO/μL and her serologies were notable for positive antihistone antibodies, positive perinuclear antineutrophil cytoplasmic antibodies, positive ANA 1:1280, anti-myeloperoxidase (MPO) antibodies, negative anti-dsDNA, and negative serine protease 3.

Within 48 hours of consultation, the patient went into shock, developed a fever, decompensated, and was transitioned to comfort care and passed away peacefully.

In postmortem examination, skin from 2 discrete sites demonstrated identical histologic features: zones of epidermal and dermal necrosis with blister formation, hemorrhage, small- and medium-vessel vasculitis with fibrinoid necrosis, edema, and a brisk perivascular inflammatory infiltrate. The inflammation was composed of “cryptococcoid” mononuclear cells with prominent perinuclear clearing as well as scattered neutrophils and lymphocytes. Immunohistochemical staining for myeloperoxidase highlighted a majority of the mononuclear infiltrate and associated inflammatory debris, supporting a myeloid lineage for these cells.

## Discussion

Hydralazine-associated vasculitis is a rare phenomenon that presents differently than hydralazine-associated SLE, often with more severe cutaneous manifestations.^3^ Reported risk factors include older age of onset, female gender, and underlying autoimmune disorders. The laboratory findings of a positive serum perinuclear antineutrophil cytoplasmic antibodies and anti-MPO in the absence of anti-dsDNA are consistent with hydralazine-associated vasculitis, whereas the finding of elevated ANA and anti-histone antibodies is more commonly associated with hydralazine-associated SLE.^2^ There are reported cases of overlap between the 2.^1^

This patient’s pathergy is of particular note because it is more commonly associated with neutrophilic dermatosis, and is not thought to be a classic finding in vasculitis or lupus. One exception is Behçet’s disease, a neutrophilic vasculitis that demonstrates pathergy. There were no identifiable reports of hydralazine-associated vasculitis and pathergy in the literature, but it was a prominent feature of this patient’s rash. The histologic findings of vasculitis and a Sweet syndrome-like eruption with “cryptococcoid” myeloid cells matches what has been previously been reported in the setting of hydralazine-associated vasculitis; however, the previous case reports have lacked pathergy.^1^ Multiple reports have found shared histologic features between hydralazine-associated vasculitis and SLE with Sweet syndrome.^1^^,^^2^^,^^4^ Given the rarity of this hydralazine-associated vasculitis, as well as its histologic overlap with Sweet syndrome, pathergy may be more commonly seen in this condition than is currently reported and further investigation is warranted.

Multiple theories have been proposed regarding the mechanism through which hydralazine triggers autoantibody formation. One suggested mechanism is that hydralazine is metabolized by MPO, producing reactive intermediate metabolites that accumulate in neutrophils, eventually causing apoptosis and spillage of normally sequestered antigens.^5^ These cytotoxic metabolites may also lead to abnormal breakdown of chromatin in at-risk individuals, producing an autoimmune response against histone-DNA complexes. Another theory is that hydralazine may serve as a hapten and join with MPO, inducing an immune response against a hydralazine-MPO complex. This response can result in the creation of anti-MPO in susceptible individuals.^6^ Interestingly, this patient had multiple listed prior drug allergies because of her HaT.

Incidentally, although of note to dermatologists, this patient also has HaT confirmed by gene copy number analysis. HaT is an autosomal dominant genetic trait with an estimated prevalence of 5.7% in the United States.^7^ The majority of individuals with the genetic trait are asymptomatic, but it can increase the frequency and severity of immediate hypersensitivity reactions in up to one third of affected individuals. It is unclear whether this patient’s HaT played any role in her cutaneous findings, but skin findings in HaT remain an underreported in the dermatology literature and may be relevant to dermatologists.

## Conclusion

We report a unique case of diffuse hydralazine-associated bullous vasculitis with features of pathergy, expanding the spectrum of cutaneous morphologic signs in this entity.

## Conflicts of interest

None.
